# Actionable clinical decisions based on comprehensive genomic evaluation in asymptomatic adults

**DOI:** 10.1002/mgg3.154

**Published:** 2015-05-06

**Authors:** Nir Pillar, Ofer Isakov, Daphna Weissglas-Volkov, Shay Botchan, Eitan Friedman, Nadir Arber, Noam Shomron

**Affiliations:** 1 Faculty of Medicine, Tel Aviv UniversityTel Aviv, 69978, Israel; 2 The Susanne Levy Gertner Oncogenetics Unit, The Danek Gertner Institute of Human Genetics, Chaim Sheba Medical CenterTel-Hashomer, Israel; 3 The Integrated Cancer Prevention Center, Tel Aviv Medical Center, Tel Aviv UniversityTel Aviv, Israel

**Keywords:** Exome sequencing, clinical decision, genome, genomic

## Abstract

Whole-exome sequencing (WES) arises as a new approach in diagnosing individuals affected by multigenic and complex phenotypes. Herein, we aim to examine whether WES is useful in screening asymptomatic individuals for actionable interventions, which has not yet been established. Twenty-five healthy adults underwent WES, bioinformatics, and manual curation of their exomes. Six participants (24%) harbored significant, management-changing variants in cancer predisposition genes, American College of Medical Genetics, and genomics reportable cardiac diseases and pharmacogenomic biomarkers that have led to clinical recommendations and interventions. Furthermore, more than 80% of the participants (21) carried 1–3 genetic variants with an associated clinical guideline for an altered drug dosing or administration based on the FDA’s table of pharmacogenomics. These results support WES potential not only to answer specific diagnostic questions presented by the relevant personal and/or family history but also to uncover clinically important genetic findings unrelated to the primary indication for sequencing.

## Introduction

Next-generation sequencing (NGS) has revolutionized both medical research and diagnostics (Koboldt et al. 2013). This innovative technology enabled generation of a multitude of DNA sequences and genomic data that is measured in Exabytes with a short turnaround time and low error rates. Applying this technology in the research arena helped in shedding light on the genetic basis of rare familial syndromes (Boycott et al. 2013), and discovering novel mutations and various gene expression regulatory mechanisms (Li et al. 2014). In clinical practice, whole-exome sequencing (WES) facilitates a new approach in diagnosing individuals affected by multigenic and complex phenotypes such as cancer (Tang et al. 2013), autism (Buxbaum et al. 2012), and heart diseases (Yang et al. 2014a). NGS reduce time and resource-consuming diagnostic procedures by sequencing in parallel, rather than sequentially, all known genes that are relevant to the studied phenotype. It enables physicians to concomitantly search for multiple mutations and objectively assess their risk for developing a variety of phenotypes, an assessment that may have a discernable effect on clinical recommendations and in particular assist in early detection and primary prevention of cancer, prenatal diagnosis, and pharmacotherapy. In addition, algorithms for identifying responders and nonresponders to medications, avoiding adverse events, and optimizing drug dosage and/or intervals based on genomic data are being explored (Bielinski et al. 2014) and over 100 drugs are currently listed on the U.S. Food and Drug Administration (FDA) pharmacogenomics biomarker list (Research n.d.).

As sequencers improve and the cost of WES continues to drop, this innovative tool stands on the verge of becoming an integral part of clinical diagnostics. In apparently healthy individuals, WES can uncover potential significant clinical genetic findings. However, questions as to the impact feasibility and ethical issues pertaining to the clinical utility of WES and actionable steps in asymptomatic individuals still remain unresolved.

Recently, the American College of Medical Genetics and genomics (ACMG) published guidelines and recommendations for which are the genes where pathogenic mutations are detected in the course of clinical WES should be reported to the patients even if there is a lack of relevant family history. Currently, it is recommended to include mutations in 56 genes that are associated with highly penetrant, monogenic disorders (Green et al. 2013). These guidelines, raised ethical and legal dilemmas and public concerns whether the public should be screened for these mutations (Pillar et al. 2014), and whether these incidental findings are indeed actionable at all ages. From the public’s standpoint, nearly 100% of the general public surveyed expressed an interest to have the significant findings with clinical implication conveyed to them (Strong et al. 2014), and more than 85% of medical geneticists surveyed thought that incidental NGS findings should be reported and discussed with the genotyped individuals (Yu et al. 2014).

Studies assessing the feasibility of implementing NGS for clinical diagnosis of individuals clinically suspected for having a genetically based condition have previously been reported. In 814 consecutive patients with undiagnosed, suspected genetic conditions, WES yielded an overall molecular diagnosis rate of 26% (Lee et al. 2014). Another study focused primarily on the pediatric age group that encompassed 2000 patients with suspected genetic disorders reported a similar genetic diagnosis rate (Yang et al. 2014b). A retrospective study conducted on the National Heart, Lung, and Blood Institute Exome Sequencing Project database of 1000 exomes, of randomly selected European and African descent participants revealed 2–3% prevalence of high-penetrance, actionable pathogenic or likely pathogenic (Dorschner et al. 2013). Only one of 12 healthy individuals who underwent WES for general screening harbored an actionable germline genetic variant (Dewey et al. 2014).

The main objective of this study was to evaluate the feasibility and yield of applying NGS in screening asymptomatic individuals for actionable findings that could affect disease prevention, risk reduction, and early detection.

## Patients and Methods

Study participants (*n* = 25), all adults (age 25 and above), who expressed interest in sequencing their exome, were recruited from the Integrated Cancer Prevention Center at Tel Aviv Medical Center, Tel Aviv, between 2012 and 2013. None of the study participants had a known inherited disorder prior to testing. All study participants received medical counseling from a certified geneticist (EF) and their primary care physician (NA). All participants signed an informed consent. The local ethics committee and the Ministry of Health in Israel approved the study.

### Library preparation, exome capture, and sequencing

Genomic DNA was isolated from peripheral blood leukocytes. Library preparation for NGS was performed according to the TruSeq (Illumina, San Diego, CA) sample-preparation protocol. DNA libraries were then hybridized to exome-capture probes with NimbleGen SeqCap EZ Human Exome Library, version 2.0 (Roche NimbleGen, Madison, WI). Exome-enriched libraries were sequenced on the HiSeq 2000 (Illumina) with an average coverage of 80-fold per each sample.

### Variant calling and prioritization

Sequence reads were aligned to the reference human genome (GRCh37/hg19), using the Burrows Wheeler Aligner (BWA) (Li and Durbin 2009). Variants were called following the Genome Analysis Toolkit (GATK) (McKenna et al. 2010) best practices. Briefly, duplicate reads were marked using Picard (http://picard.sourceforge.net). Reads were realigned around detected insertions and deletions (indels) and base qualities recalibrated using GATK. Variant calling was performed using the GATK UnifiedGenotyper tool. Variants with low base or mapping qualities, demonstrating strand bias, aberrant read position distribution or reference versus alternate quality score discrepancy were marked and filtered from subsequent analysis. Variants were annotated using ANNOVAR (Wang et al. 2010) with frequency information gathered from dbSNP138 (Sherry et al. 2001), European and general population from the 1000 genomes project (http://www.1000genomes.org/), NHLBI Exome Sequencing Project (http://evs.gs.washington.edu/EVS/) and a personal database of 100 Ashkenazi Jews exomes. Insertions and deletions (Indels) found adjacent to homopolymers longer than 5 bases or repeats were marked and were not considered as true candidates. For monogenic diseases diagnosis, variants with allele frequencies higher than 1% in any of the databases were considered prevalent and were excluded from downstream analysis. For polygenic diseases and pharmacologic-related variants, variants with frequency higher than 10% were excluded. Variant severity was predicted using 10 different prediction tools gathered by dbNSFP (Liu et al. 2013). A variant was considered to be deleterious if more than half of the prediction tools mark it as such. Variants were prioritized by combining the aforementioned annotations with information regarding their affected gene. Gene disease associations were collected from OMIM (http://omim.org/), Orphanet (http://www.orpha.net), and the Human Phenotype Ontology (Robinson and Mundlos 2010). Tissue expression and developmental stages were collected from Uniprot knowledge base (Magrane and Consortium 2011). Genic intolerance to functional variation (RVIS) was retrieved from (Petrovski et al. 2013). Combination of variant and gene annotation data was performed using in-house scripts. Coverage was calculated using the Ensembl coding sequence regions.

### Cancer- and cardiovascular-related variants

ACMG statement on incidental findings in clinical genomics and COSMIC list of germline mutations in cancer (Forbes et al. 2015) were used for reportable genes selection. Sequence variants in cancer and cardiovascular disorders associated genes were predicted for their deleterious effect using 10 online algorithms combined by dbNSFP. Each gene alteration was given a rank ranging 0–10 according to the number of tools predicted its harmful effect. Variants scored above 5 were considered to be deleterious and were reported for further manual curation.

### Genetic drug-response predictions

Genotype data at loci with reported clinical drug-response predictions were intersected with annotations cataloged in the PharmGKB knowledge base (https://www.pharmgkb.org/). Drug-response associations with level of evidence of 1B or higher (replicated associations, implemented in a major health system or pharmacogenomics research network site, or endorsed in a medical society guideline) were reported and further crossed with the FDA’s table of pharmacogenomic biomarkers in drug labeling and manually reviewed.

### Manual curation

The multidisciplinary team composed of a genetic counselor (E.F.), an internist and gastroenterologist (N.A.), a pharmacology specialist (M.B.), and a bioinformatics specialist (N.S.) manually reviewed the variants. When needed (Mati Berkovitz), the team sought the input of the appropriate specialist. The team reviewed all variants predicted to be deleterious by combining computational algorithms. This consortium performed literature review using several databases (PUBMED, OMIM, HGMD and GeneCards) and classified candidate variants according to variant and gene-level evidence for pathogenicity and description of the clinical phenotype for reporting.

### Validation

All participants underwent Sanger sequencing of *APC* (OMIM 611731), targeted for I1307K and E1317Q variants. 100% concordance was noted between these and exome analysis. In addition, 1–2 clinically relevant mutations, selected by a multidisciplinary team per each participant were validated via Sanger sequencing with 95% concordance.

## Results

### Participant characteristics

All participants were Caucasians of Jewish origin, the median age was 52 (35–74) years and 60% were males. Demographics of study participants are presented on Table S1. Extensive personal and family history was obtained from all participants.

### Genetic analyses

Each participant harbored an average of 969,737 coding variants (472,228–1,806,428), of which 235 (197–313) were defined as rare and severe, and 23 (1–86) were novel single-nucleotide variants (Table S2). Two top key variants predicted to be deleterious with respect to personal risk and carrier status for inherited diseases were selected for validation using Sanger sequencing with a 100% concordance. The validity of the WES was further assessed in all the subjects for the I1307K and E1317Q APC variants.

#### Cancer-related genes

An average of 1.4 cancer-related genes with predictable deleterious variants were noted per participant (range 0–3 genes). Three harbored more than one cancer predisposition gene mutation.

Breast cancer-related variants – *BRCA1* (OMIM 113705), *BRCA2* (OMIM 600185), and *PALB2* (OMIM 610355 were present in four patients (16%); Lynch syndrome-related gene sequence alterations – *MLH1* (OMIM 120436)*, MSH2* (OMIM 609309), *MSH6* (OMIM 600678)*, PMS2* (OMIM 600259)*, and PMS1* (OMIM 600258 were detected in six patients (24%).

#### Cardiovascular-related genes

**Figure 1:**
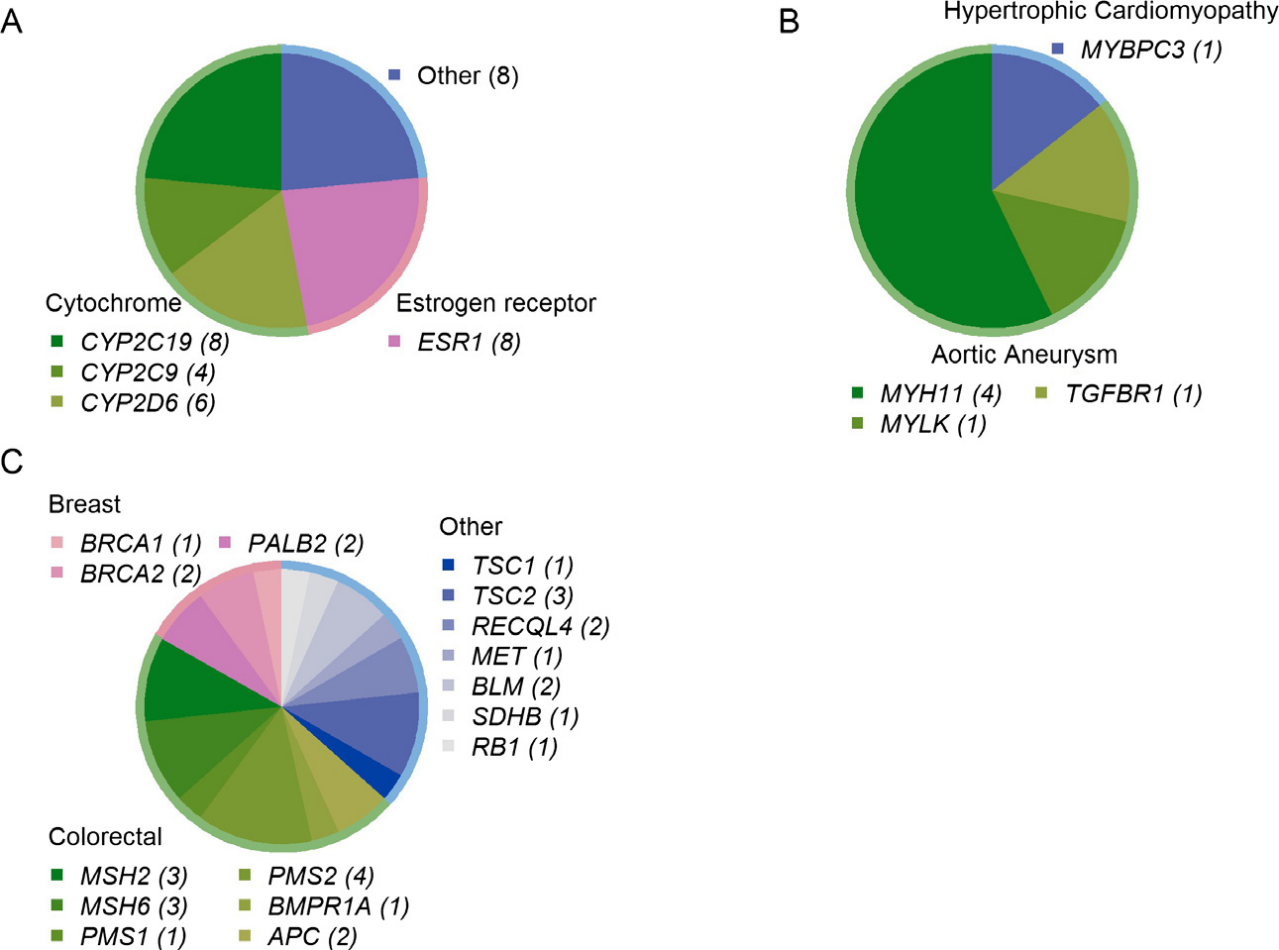
Deleterious variants in pharmacogenomics, cardiovascular, and cancer genes. (A) Pharmacogenomics-related genes (B) Cardiovascular-related genes (C) Cancer-related genes. The bracketed numbers represent number of individuals with computationally predicted deleterious variants in each gene.

Six patients (24%) harbored deleterious variants in CVS disorders-related genes. Thoracic aortic aneurysms-related variants were detected in five patients (20%) and one patient (5%) had hypertrophic cardiomyopathy-related variant. Variants predicted to be deleterious in cancer, cardiovascular, and pharmacogenomics genes are presented in Figure1.

### Clinically significant findings

**Figure 2:**
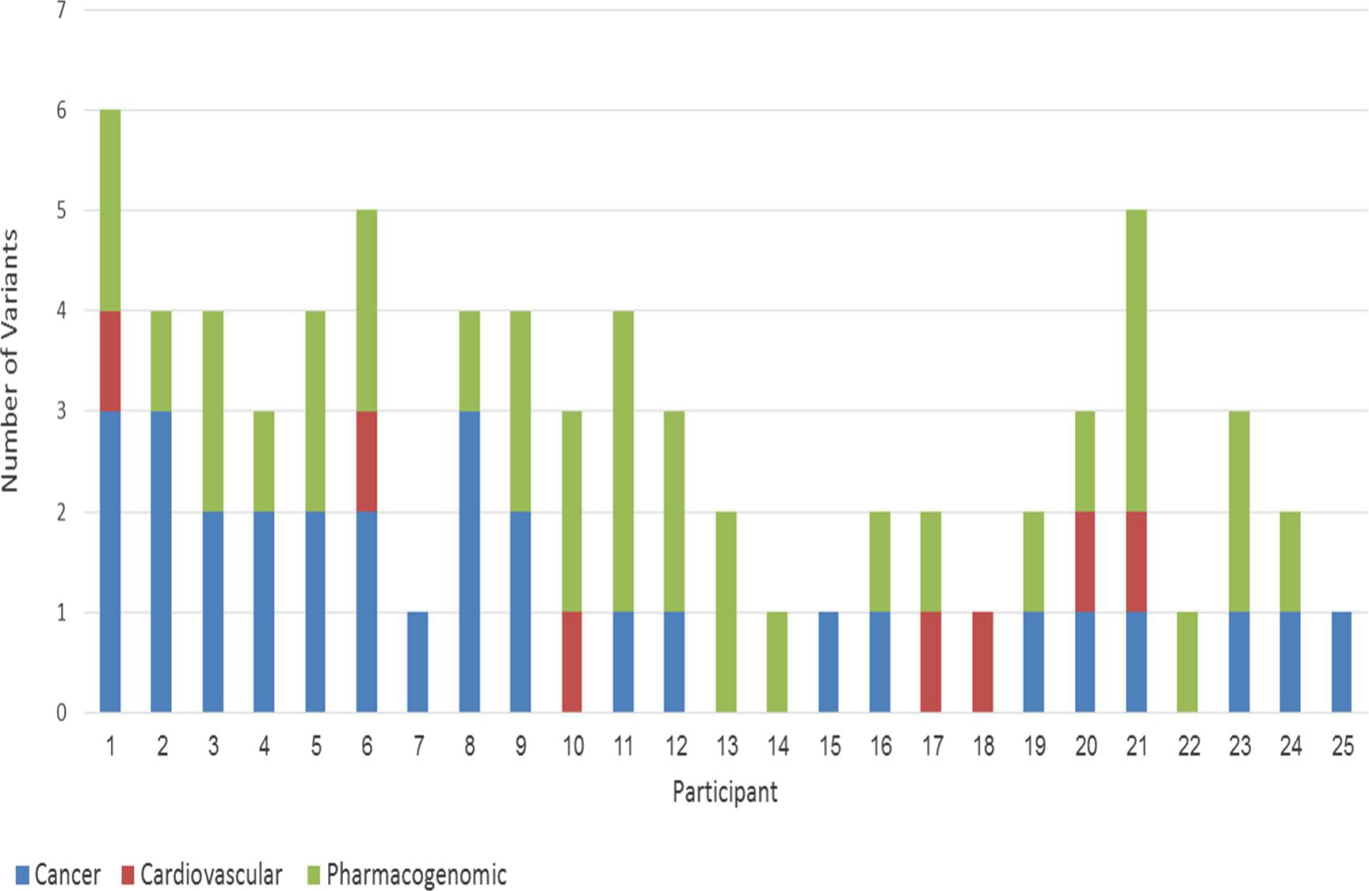
Dispersion of variants per participant in cancer, cardiovascular, and pharmacogenomics categories. Each number on the horizontal axis represents individual underwent WES. The vertical axis represent predicted deleterious variants in cancer, cardiovascular, and pharmacogenomics.

After genetic variants discovery in all participants was concluded and deleterious variants were flagged and validated, a multidisciplinary team assessed all seemingly deleterious variants. The dispersion of variants predicted to harbor deleterious mutations are displayed in Figure2.

### Six participants (24%) harbored significant, management-altering variants

- A 55-year-old male with severe ischemic heart disease (three coronary angioplasties) who is treated with Clopidogrel. A nonsynonymous single-nucleotide variation in *CYP2C19* (OMIM 124020, rs4244285) is reportedly associated with reduced efficacy of Clopidogrel treatment. Hence, Clopidogrel dosage was increased.
- A 43-year-old female with no family history of breast or ovarian cancer, but a father was diagnosed with pancreatic cancer at age 67 years. A frameshift deleterious mutation – a 19 nucleotide deletion in exon 11 in *BRCA2* (rs80359550) was found. At genetic consultation meticulous breast cancer screening by MRI alternating with breast mammograms every 6–12 months as well as risk reduction bilateral salpingo-oophorectomy (RRBSO) were recommended.
- A 55-year-old male with two tubulovillous adenomas (7 mm in the transverse colon and 5 mm in the cecum) were resected during routine screening colonoscopy at age 50 years. Two nonsynonymous single-nucleotide variations in *MSH2* (rs202026056) and *MSH6* (rs376220212) were detected. Recommendation to adhere to a Lynch syndrome surveillance scheme was made.
- A 54-year-old physically active male with an incidental finding of dilated ascending aorta that was stable for 7 years. He was found to harbor a nonsynonumous single-nucleotide variation in *MYH11* (OMIM 160745, rs146228576). The patient was prescribed *β*-blockers and advised to decrease exertion physical activity.
- A 48-year-old female without personal or family history of gastrointestinal malignancy. A nonsynonymous single-nucleotide variation in *BMPR1A* (OMIM 601299, rs140592056) was detected and led to recommendation to perform gastroscopy and capsule endoscopy with the next colonoscopy procedure.
- A 74-year-old woman who underwent removal of recurrent advanced adenomas on three sequential colonoscopies. A nonsynonymous single-nucleotide variation in *PMS1* (rs142159998) gene was detected and assessed as potentially related to the phenotype and led to the recommendation to continue the appropriate Hereditary nonpolyposis colorectal cancer (HNPCC) surveillance. Her daughter, 42-year old, does not carry this mutation; hence she was switched from an HNPCC surveillance program to normal colorectal cancer (CRC) screening program.

Fourteen additional participants were found to carry genetic variants categorized as disease causing in the Human Gene Mutation Database (HGMD) that the medical consulting team had reclassified as reportable variants of uncertain significance.

## Discussion

WES is increasingly used in clinical medicine and has the potential not only to answer specific diagnostic questions presented by the relevant personal and/or family history but also to uncover clinically important genetic findings unrelated to the primary indication for sequencing.

This study aimed to evaluate the feasibility and yield of applying WES as a screening tool for the general, healthy population. We used an “upside down” approach to genetic-based diagnosis, where subjects were initially screened for pathogenic mutations followed by manual curation and disclosing actionable results to the primary physician and participants with specific recommendations whenever appropriate. Six of the participants (24%) manifested inherited disease-risk variants that are likely pathogenic and have led to significant alterations in the clinical recommendations and treatment. More than 80% of the participants carried 1–3 genetic variants with an associated clinical guideline for an altered drug dosing or administration based on the FDA’s table of pharmacogenomics.

Despite major improvements in analyzing and interpreting the genomic data, processing of WES data into actionable decisions is complex. From the clinical geneticists’ perspective the ability to generate a large amount of genetic data for each counseled individual in a single experiment and the need to interpret these data into concrete, clinically relevant recommendations to be disclosed to the participant is a major undertaking. Unlike “classical” genetic testing that focuses on one or a few genes in the context of a specific personal and family history, WES provides a new framework of reference, and emphasizes the need to generate new adoptable guidelines that would ensure that all possible benefits as well as confidentiality are adequately maintained. Manual curation and biological validation remain a crucial step in results accuracy and assigning putative clinical significance. The number of reportable genetic findings from WES is likely to differ with sequencing expertise, variation in pathogenicity classifications and manual validation methodology, as well as the often unknown or unreported family history. Furthermore, the entire spectrum of clinically significant mutation identification is not satisfied by exome sequencing alone at present. Structural variants are not reliably identified and the overall exome coverage varies between genes and exons, so that some genome coding areas are not consistently covered at a read depth that is sufficient for a comprehensive, clinically significant assignment of genetic variants (Wang et al. 2013). Taken together, limitations of WES combined with our incomplete knowledge of the inherited basis of human disorders (>150,000 pathogenic variants listed in the HGMD) are all major obstacles for variant interpretation (Gonzalez-Garay et al. 2013).

The clinical interpretation of NGS data yet requires a multidisciplinary team in classifying the variants passing bioinformatics filtering. In this study, more than 80% of variants predicted to be harbor deleterious mutations were reclassified as polymorphic after manual curation. Review of results, including time required for literature search (finding literature cataloged in mutation databases and performing independent PubMed and Google searches for the genetic variant and gene), required a median of 45 min (range, 12–172 min) per genetic variant.

One of the major strengths of this study is the comprehensive multidisciplinary follow up of all cases over 10 years in a single tertiary medical center. This enabled thorough documentation of the personal and family phenotype as these evolved, facilitating correlation of the phenotype with the WES generated genotype and allowed to check additional family members according to the genomic findings.

However, given the small study sample, of mostly Jewish Ashkenazim from a single medical center in Israel, we have limited ability to generalize the results to other, larger and ethnically diverse populations.

Currently, genomic risk information disclosure will need to be conveyed by a trained team of physicians and genetic counselors. Until accepted guidelines and generally accessible and reliable data sources exist for clinical interpretation of genetic variants this approach should be adopted. However, as more evidence accumulates screening asymptomatic individuals, still in its infancy appears as a very promising technology in the near future.

In conclusion, this study showed that about a quarter of 25 healthy adult Jewish individuals harbored significant, actionable variants in cancer predisposition genes, ACMG reportable mutations for cardiac diseases and pharmacogenomics biomarkers, variants that have led to clinical recommendations, actions and surveillance.

## Conflict of Interest

The authors declare that they have no conflict of interest.

## Ethical Approval

All procedures performed in studies involving human participants were in accordance with the ethical standards of the institutional and/or national research committee and with the 1964 Helsinki declaration and its later amendments or comparable ethical standards.

## Informed Consent

Informed consent was obtained from all individual participants included in the study.

## Supporting Information

**Table S1.** Demographics of study participants. **Table S2.** Sequencing results for study participants.
