# Supplementary material for: Actionable clinical decisions based on comprehensive genomic evaluation in asymptomatic adults
Source: Mol Genet Genomic Med. 2015 May 6;3(5):433–9. doi: 10.1002/mgg3.154 (PMC4585451; doi:10.1002/mgg3.154)
Supplement: Supplementary file 1 [file mgg30003-0433-sd1.doc]

Table s1 - Demographics of study participants

| **Participant** | **Gender** | **Age** | **Place of birth** | **Ethnicity** |
| --- | --- | --- | --- | --- |
| 1 | M | 54 | England | Ashkenazi |
| 2 | F | 38 | Israel | Syrian/Ashkenazi |
| 3 | F | 41 | Israel | Ashkenazi |
| 4 | F | 74 | Romania | Ashkenazi |
| 5 | F | 42 | Israel | Ashkenazi |
| 6 | M | 46 | Israel | Syrian/Iraq |
| 7 | F | 35 | Israel | Ashkenazi |
| 8 | M | 53 | Israel | Ashkenazi |
| 9 | M | 58 | Israel | Ashkenazi |
| 10 | M | 56 | Israel | Ashkenazi |
| 11 | F | 44 | Colombia | Ashkenazi |
| 12 | M | 52 | Israel | Ashkenazi |
| 13 | M | 39 | Israel | Ashkenazi |
| 14 | F | 54 | Israel | Ashkenazi |
| 15 | M | 53 | USA | Ashkenazi/Sepharadi |
| 16 | M | 49 | Israel | Ashkenazi |
| 17 | F | 48 | Israel | Ashkenazi |
| 18 | M | 55 | Israel | Ashkenazi |
| 19 | F | 42 | Israel | Ashkenazi |
| 20 | M | 52 | Israel | Ashkenazi |
| 21 | M | 53 | Israel | Ashkenazi |
| 22 | M | 60 | Israel | Yeman |
| 23 | M | 53 | Israel | Ashkenazi |
| 24 | F | 48 | Uruguay | Ashkenazi |
| 25 | M | 54 | Israel | Ashkenazi |

Table s2- Sequencing results for study participants

| **Participant** | **Rare** | **Rare exonic** | **Rare severe** | **Very rare exonic** | **Very rare severe** | **Novel exonic** | **Novel severe** | **Total** |
| --- | --- | --- | --- | --- | --- | --- | --- | --- |
| **1** | 36884 | 1476 | 254 | 695 | 140 | 221 | 36 | 1295627 |
| **2** | 34676 | 1514 | 231 | 712 | 134 | 271 | 47 | 1126281 |
| **3** | 34866 | 1491 | 226 | 645 | 123 | 217 | 39 | 1092977 |
| **4** | 22369 | 1429 | 227 | 675 | 124 | 226 | 42 | 768922 |
| **5** | 36265 | 1439 | 234 | 676 | 129 | 236 | 41 | 1258860 |
| **6** | 32861 | 1507 | 259 | 656 | 133 | 241 | 41 | 1096893 |
| **7** | 14970 | 1305 | 218 | 590 | 117 | 153 | 28 | 472288 |
| **8** | 31840 | 1502 | 224 | 677 | 124 | 272 | 43 | 1120451 |
| **9** | 25103 | 2008 | 313 | 798 | 145 | 289 | 44 | 935993 |
| **10** | 37022 | 1493 | 227 | 672 | 123 | 221 | 37 | 1305717 |
| **11** | 20918 | 1566 | 247 | 848 | 148 | 449 | 86 | 715841 |
| **12** | 26340 | 1384 | 225 | 620 | 121 | 216 | 39 | 866336 |
| **13** | 14312 | 1289 | 244 | 527 | 133 | 10 | 1 | 696585 |
| **14** | 14601 | 1251 | 215 | 421 | 92 | 13 | 4 | 791771 |
| **15** | 31692 | 1254 | 231 | 408 | 99 | 15 | 5 | 1806428 |
| **16** | 13161 | 1220 | 204 | 399 | 90 | 15 | 4 | 623771 |
| **17** | 12959 | 1265 | 223 | 394 | 93 | 8 | 3 | 648977 |
| **18** | 11737 | 1156 | 205 | 405 | 103 | 10 | 3 | 500971 |
| **19** | 16479 | 1289 | 243 | 506 | 130 | 11 | 3 | 920315 |
| **20** | 15440 | 1206 | 235 | 430 | 112 | 13 | 4 | 835713 |
| **21** | 16922 | 1390 | 241 | 542 | 120 | 13 | 2 | 885189 |
| **22** | 16375 | 1238 | 197 | 406 | 93 | 12 | 4 | 945394 |
| **23** | 17873 | 1500 | 274 | 714 | 157 | 14 | 6 | 858988 |
| **24** | 27149 | 1210 | 235 | 397 | 93 | 14 | 4 | 1687679 |
| **25** | 23584 | 1542 | 273 | 531 | 148 | 14 | 6 | 985462 |
| **Average** | 23456 | 1397 | 236 | 574 | 121 | 127 | 23 | 969737 |
